# Food sources of fiber and micronutrients of concern among infants and young children in Lebanon: a national cross-sectional study

**DOI:** 10.1186/s12887-024-04535-2

**Published:** 2024-01-19

**Authors:** Fatima Al Zahraa Chokor, Nahla Hwalla, Farah Naja, Lara Nasreddine

**Affiliations:** 1 Department of Public Health, College of Health Sciences, QU Health, Qatar University, P.O. Box 2713, Doha, Qatar; 2 Department of Nutrition and Food Sciences, Faculty of Agricultural and Food Sciences, American University of Beirut, P.O. Box 11-0236, Beirut, Lebanon; 3 Department of Clinical Nutrition and Dietetics, College of Health Sciences, Research Institute of Medical and Health Sciences (RIMHS), University of Sharjah, Sharjah, United Arab Emirates; 4 Faculty of Agricultural and Food Sciences, American University of Beirut, Beirut, Lebanon

**Keywords:** Infants, Children, Lebanon, Food sources, Fiber, Micronutrients

## Abstract

**Background:**

Intakes of fiber, iron, zinc, calcium, vitamin D, vitamin A, and folate were shown to be low in a substantial proportion of infants and children in Lebanon. The study aims to identify the top food sources of fiber, iron, zinc, calcium, vitamin D, vitamin A, and folate amongst infants and young children in Lebanon and to evaluate the evolution of food sources of these nutrients from the beginning of the complementary feeding journey up until the age of 47.9 months.

**Methods:**

A national cross-sectional survey was conducted in 2012 as part of the “Early Life Nutrition and Health in Lebanon” project using stratified cluster sampling. Dietary intakes for infants and young children aged 6-47.9 months (*n* = 763) were assessed using 24- Hour Dietary Recall. Food items were categorized into food groups and the percent contribution of each food group to nutrient intakes was determined to identify the top food sources of fiber and selected micronutrients for three age groups: 6-11.9 m (infants), 12-23.9 m (toddlers), and 24-47.9 m (preschoolers).

**Results:**

The top food source of fiber was vegetables among children aged 6-47.9 months. Among infants and toddlers, infant/young child formula was the main contributor to iron, zinc, calcium, vitamin D, vitamin A, and folate intakes. Baby cereals also contributed to around 14% of iron intakes among infants. Among preschoolers, meat and fish contributed to 13% of iron intakes and 29% of zinc intakes, while cow’s milk was the major contributor of calcium (41%), vitamin D (81%) and vitamin A (25%) intakes. Sweetened beverages and sweet bakery were also ranked among the major food sources contributing to substantial intakes of key nutrients, including fiber, iron, zinc, calcium, vitamin A, and folate among infants, toddlers, and preschoolers.

**Conclusions:**

In addition to milk sources, vegetables, beans and legumes, breads, meats, and rice and pasta, sweet bakery and sweetened beverages have contributed to intakes of key nutrients from early ages. This calls for implementing initiatives and designing approaches to support nutrition education and improve nutrient intakes in infancy and early childhood.

## Background

Rapid demographic and epidemiologic changes brought by urbanization, modernization, and technological development have escalated the nutrition transition in many Eastern Mediterranean countries [1], including Lebanon [2]. Young children may be amongst the most vulnerable population groups to the ongoing nutrition transition, as their diets are becoming increasingly energy-dense while being poor in fiber and several micronutrients [3]. This is of concern given that adequate nutrition in early childhood is crucial for optimal physical, motor and cognitive development [4], and for setting the stage for healthy adolescence/young adulthood [4].

Previous studies conducted in Lebanon have reported a triple burden of malnutrition (underweight, nutrient deficiencies and overweight/obesity) among infants and young children, and the intake of several micronutrients has been described as inadequate in this age group [2, 4, 5]. The intakes of iron, zinc, calcium, vitamin D, vitamin A, and folate were shown to be low in a substantial proportion of infants and children aged 0–4 years [5]. In specific, intakes of iron and zinc were inadequate in 45.3% and 21.6% of Lebanese infants aged 6-11.9 months, respectively while vitamin D and calcium were below the Estimated Average Requirement (EAR) among 84.7% and 44.6% of Lebanese toddlers, respectively [5]. Inadequate intakes of micronutrients may contribute to the development of overt deficiencies, leading to detrimental health outcomes on the short-term as well as the long-term [6]. For instance, iron deficiency can adversely affect cognitive functions and psychomotor development, while also inducing or exacerbating deficiencies of other essential nutrients [7]. Many studies have identified zinc deficiency as a key contributor to stunting in children [8–10] in addition to being a risk factor for the manifestation of malnutrition, diarrhea, pneumonia and impaired wound healing [9]. Vitamin D deficiency and low dietary intakes of both vitamin D and calcium may lead to the establishment of rickets in children [11]. Vitamin A plays a key role in the regulation of several vital physiological processes throughout the life cycle and its deficiency is the leading cause of preventable blindness among young children, while also contributing to immune dysfunction, frequent infections, and improper growth processes [12–14]. Folate is also known to play a crucial role in optimal neural function and repair, and its deficiency is associated with the establishment of several neurodevelopmental disorders [15]. Available studies also suggest that young children in Lebanon do not meet the recommended intake levels of fiber [5], an important nutrient for the functioning of the gastrointestinal tract (providing bulk to the stool, preventing constipation), the development of a healthy gut microbiome, and the prevention of excessive weight gain and several cardio-metabolic abnormalities [16, 17].

Food sources of micronutrients and fiber may differ between countries, as a reflection of the cultural dietary practices. It is in this context that we have conducted this study, with the aim of (1) identifying the top food sources of fiber, iron, zinc, calcium, vitamin D, vitamin A, and folate amongst infants and young children in Lebanon and (2) evaluating the evolution of food sources of these nutrients from the beginning of the complementary feeding journey up until the age of 47.9 months. The analyses undertaken in this study were conducted based on the Feeding Infants and Toddlers Study (FITS) protocol [18]. The study findings will contribute to a better understanding of the main food sources of key nutrients in an Eastern Mediterranean country and hence to the development of culture-specific interventions aimed at improving the nutritional adequacy of diets in early childhood.

## Methods

### Study population

The “Early Life Nutrition and Health in Lebanon” (ELNAHL) project is a cross-sectional study based on a national survey of a representative sample of infants and young children, conducted in Lebanon in 2012. The detailed study design has been previously published elsewhere [19, 20]. In brief, households were considered as the primary sampling units in this survey. The selection of households was based on a stratified cluster sampling strategy, with the strata being the six Lebanese governorates and the clusters being selected further at the level of districts. In each district, the selection of households was performed according to a probability proportional to size approach, whereby a higher number of participating households was drawn from districts that are more crowded; the selection of households was carried out using systematic sampling. To be eligible to participate in the survey (inclusion criteria), households had to include a mother and a child aged 5 years or less. Of the 1194 eligible households that were contacted, 1029 agreed to participate in the survey, with a response rate of 86%. Exclusion criteria included non-Lebanese children and mother pairs, children born preterm (< 37 weeks), or children who had any chronic disease, inborn error of metabolism, or physical malformation that could alter their dietary intake or body composition [19]. Children who were reported by their mother as being ill during the past 24 hours (i.e. on the day that would be covered during the dietary intake) were also excluded from the study. For the present study, data pertinent to infants and young children aged between 6 and 47.9 months were considered (*n* = 763).

### Data collection

Data collection was performed in the household setting through face-to-face interviews with the mothers. Trained research nutritionists conducted the data collection, using an age-specific multi-component questionnaire [19]. The study was performed according to the guidelines specified by the Declaration of Helsinki and the study protocol was approved by the Institutional Research Board, American University of Beirut (Protocol number NUT.LN.13). Written informed consent was obtained from all participating mothers prior to enrollment in the study.

### Dietary intake assessment

Trained nutritionists performed the dietary intake assessment using the United States Department of Agriculture (USDA) Multiple Pass 24- Hour Dietary Recall (24-HR) approach, with mothers acting as proxies [21]. In the case where another caretaker shared the responsibility of child feeding, the mother consulted directly with him/her for additional information/clarification pertinent to the child’s dietary intake. The specific steps that were adopted during the dietary interview included: (1) quick food list recall, (2) forgotten food list probe, (3) time and occasion at which foods were consumed, (4) detailed overall cycle and (5) final probe review of the foods consumed.

### Dietary data analysis

The Nutritionist Pro software (version 5.1.0, 2014, First Data Bank, Nutritionist Pro, Axxya Systems, San Bruno, CA) was used for nutrients’ intakes analyses. The food composition databases used were a combination of the USDA single food items within the Nutritionist Pro software and the food composition of the Middle Eastern region developed by the American University of Beirut (AUB) [22]. Breast milk intake was estimated based on the method described by Denney et al., 2017 [23]. Daily energy, macro and micronutrient intakes were estimated for each participating child, and nutrient intakes were compared with age-specific US Dietary Reference Intakes (DRIs) as local nutrient reference values do not exist. The percent of the population with intakes greater than the EAR or the Adequate Intake (AI ) were calculated. Fiber intake was compared with the recommended AI of 19 g for 1-3.9 years old children [24].

Recipes were disaggregated into their separate ingredients, and then food items (*n* = 614) were categorized into 60 food groups based on their nutrient profile and culinary use (Table 1). These food groups were further categorized under 10 major food categories. These categories included Grain and grain products; Fruits; Vegetables; Milk and milk products; Meats and other protein sources; Savory snacks; Sweets, sweetened beverages, and desserts; Fats and oils; Condiment and sauces; and Water and unsweetened beverages. The food grouping system was adapted from the FITS US [25] to reflect the local food culture.

**Table 1:** Food group classifications

Milk & Milk Products	Fruits	Sweets, Sweetened Beverages & Desserts
Human milk	100% Juices	Baby food cookies e.g., teething biscuits
Infant/young child (I/YC) formula	Apples	Baby food desserts
Dairy products e.g., cheese, labneh, yogurt	Apricots	Candies
Cow’s milk	100% Baby Juices	Cereal and nutrition bars
	Baby food fruits	Ices and sorbets
**Meats & Other Protein Sources**	Bananas	Ice cream, frozen yogurt, puddings
Baby food meats and legumes	Berries	Milk flavor
Egg & egg products	Citrus fruits	Syrups, préserves, Jelly
Beans & legumes	Dried fruits	Sweet bakery
Meat & fish	Grapes	Sugar-sweetened beverages (SSB)
Peanut butter, nuts & seeds	Kiwi	
Roasted nuts	Melon	**Savory Snacks**
	Mixed fruits	Corn chips
**Fats & Oils**	Nectarines	Popcorn
Butter & animal fats e.g. cream	Peaches	Potato chips
Dressings, oils & olives	Pears	
	Pineapple	**Water & unsweetened beverages**
**Grains & Grain Products**	Plums	Water
Baby cereals	Pomegranates	Unsweetened beverages
Baby finger food	Other fruits	
Breads, pita, saj		**Condiments & Sauces**
Breakfast cereals	**Vegetables**	Herbs & seasonings
Crackers, rice cakes, kaak	Baby food vegetables	Gravy & sauces
Pancakes & French toast	Potatoes (non-baby food)	
Rice & pasta	Vegetables (non-baby food)	
Other grains e.g. bulgur, quinoa, flour, oats		

### Statistical analysis

To identify the top food sources of fiber and selected micronutrients, the percent contribution of each food group to nutrient intake was calculated by determining the mean nutrient intake from each food group and expressing it as a percentage of the total dietary intake of that nutrient. Analyses were performed using Stata (StataCorp. 2019 Stata Statistical Software: Release 16. College Station, TX, USA: StataCorp LP). All food groups that contributed to at least 2% of the total dietary intake were reported, and percentage contributions were tabulated by ranked order (from highest to lowest), for 3 separate age groups: 6-11.9 months (infants), 12-23.9 months (toddlers), and 24-47.9 months (preschoolers), in line with the FITS protocol [18].

## Results

The socioeconomic characteristics, distribution of nutrient intakes, and food sources of energy among children under the age of 4 years are published elsewhere [5]. Compliance with the nutrient intake recommendations in terms of percentage above the EAR or AI (in the absence of EAR) is shown here to provide context.

### Fiber

Only 3.6% and 11.5% of toddlers and preschoolers exceeded the AI level for dietary fiber, respectively. While vegetables ranked as the second food source of fiber among infants contributing to around 13.7% of fiber intake, vegetables ranked as the top food source of fiber among toddlers and preschoolers, contributing to 15.7% and 13.7% of fiber intake, respectively. The contribution of potatoes to fiber intake became progressively higher among older children, ranging from 7.3% among infants to 10.6% among preschoolers. Likewise, breads, pita, and saj (a type of local bread) contributed to 11.0% of fiber intake among preschoolers as compared to 7.1% among infants. Grain-based foods such as breads, rice and pasta, baby cereals, and other grains (e.g. bulgur, quinoa, flour, and oats) contributed to around 17% of fiber intakes among infants. This contribution became higher in older age groups (21.9% among toddlers and 18.8% among preschoolers). The opposite was noted for fruits, whereby its contribution to fiber intake was the highest among infants, with around 28.9% of total fiber intake provided by bananas, pears, apples, and peaches compared to 11.8% from fruits among preschoolers (mainly from apples, pears, and peaches). The contribution of beans and legumes to fiber intake was also lower in the older age groups (13.3% and 10.0% among toddlers and preschoolers, respectively compared to 14.7% among infants) (Table 2).

**Table 2:** Main food contributors and their percent contribution to fiber intake among infants and young children by age group in Lebanon, 2012

% exceeding AI	Age 6-11.9 m *n* = 148		Age 12-23.9 m *n* = 222		Age 24-47.9 m *n* = 393	
	NA		3.6% > AI		11.5% > AI	
Rank	Food group	% contribution	Food group	% contribution	Food group	% contribution
**1**	Beans and legumes	14.7	Vegetables (non-baby food)	15.7	Vegetables (non-baby food)	13.7
**2**	Vegetables (non-baby food)	13.7	Beans and legumes	13.3	Breads, pita, saj	11.0
**3**	Bananas	11.4	Breads, pita, saj	12.6	Potatoes (non-baby food)	10.6
**4**	Pears	7.3	Potatoes (non-baby food)	7.8	Beans and legumes	10.0
**5**	Potatoes (non-baby food)	7.3	Rice & pasta	5.5	Sweet bakery	6.1
**6**	Breads, pita, saj	7.1	Pears	5.1	Potato chips	4.8
**7**	Apples	7.1	Bananas	4.6	Apples	4.7
**8**	Sweet bakery	4.3	Sweet bakery	4.2	Other grains	4.7
**9**	Rice & pasta	4.1	Other grains	3.8	Pears	4.7
**10**	Baby cereals	3.4	Apples	3.6	Condiments, herbs, and seasonings	3.8
**11**	Peaches	3.1	Potato chips	3.5	Peanut butter, nuts, & seeds	3.3
**12**	Other grains	2.4			Rice & pasta	3.1
**13**					Peaches	2.4
**Top contributors**:		**85.9**		**79.7**		**82.8**

### Iron

Iron intakes were found to be inadequate (< EAR) in 45.3%, 9.0%, and 11.2% of infants, toddlers, and preschoolers, respectively. The main source of iron among infants and toddlers was infant/young child (I/YC) formula, which provided 60.6% and 48.9% of iron intake, respectively. Baby cereals ranked as the second top contributor to iron intakes among infants (14.4%), while meat & fish was the second major contributor to iron intake among toddlers. In preschoolers, the top-ranking sources of iron were meat and fish followed by herbs and seasonings, providing 13.4% and 13.3% of iron intake, respectively. Beans and legumes contributed to no more than 3.2%, 4.7%, and 5.7% of iron intakes among infants, toddlers, and preschoolers, respectively (Table 3).

**Table 3:** Main food contributors and their percent contribution to iron intake among infants and young children by age group in Lebanon, 2012

% exceeding EAR	Age 6-11.9 m *n* = 148		Age 12-23.9 m *n* = 222		Age 24-47.9 m *n* = 393	
	54.7% > EAR		91% > EAR		88.8% > EAR	
Rank	Food group	% contribution	Food group	% contribution	Food group	% contribution
**1**	Infant/young child (I/YC) formula	60.6	Infant/young child (I/YC) formula	48.9	Meat & fish	13.4
**2**	Baby cereals	14.4	Meat & fish	5.9	Herbs & seasonings	13.3
**3**	Rice & pasta	3.6	Rice & pasta	5.4	Vegetables (non-baby food)	9.1
**4**	Sweet bakery	3.4	Sweet bakery	4.9	Sweet bakery	9.0
**5**	Beans and legumes	3.2	Vegetables (non-baby food)	4.8	Rice & pasta	7.4
**6**			Beans and legumes	4.7	Beans and legumes	5.7
**7**			Herbs & seasonings	4.0	Breakfast cereals	5.1
**8**			Baby cereals	3.1	Sweetened beverages	3.5
**9**			Breakfast cereals	3.0	Potatoes (non-baby food)	3.1
**10**			Sweetened beverages	2.3	Peanut butter, nuts, and seeds	3.1
**11**					Dairy products	3.0
**12**					Potato chips	2.6
**13**					Other grains	2.1
**Top contributors**:		**85.2**		**87.0**		**80.4**

### Zinc

More than 78% of infants had adequate intakes of zinc, and this proportion increased to around 93% and 96% among toddlers and preschoolers, respectively. I/YC formula was the top contributor to zinc intake among infants and toddlers, being estimated at 56.2% and 41.2%, respectively. Meat & fish ranked as the third contributor to zinc intake amongst infants (7.2%), while the contribution of this food group became higher in older age groups (17.7% in toddlers and 28.9% in preschoolers). Cow’s milk was identified as the second contributor to zinc intake in preschoolers (16.6%). Grains have also contributed to zinc intakes among infants, with 6.6%, 2.7%, and 2.4% of zinc intake being provided by baby cereals, rice & pasta, and breads, respectively. Breads as well as rice & pasta provided a higher contribution to zinc intakes among older age groups with 6.1–7.0% for breads and 4.2–3.6% for rice & pasta among toddlers and preshoolers, respectively (Table 4).

**Table 4:** Main food contributors and their percent contribution to zinc intake among infants and young children by age group in Lebanon, 2012

% exceeding EAR	Age 6-11.9 m *n* = 148		Age 12-23.9 m *n* = 222		Age 24-47.9 m *n* = 393	
	78.4% > EAR		93.2% > EAR		95.9% > EAR	
Rank	Food group	% contribution	Food group	% contribution	Food group	% contribution
**1**	Infant/young child (I/YC) formula	56.2	Infant/young child (I/YC) formula	41.2	Meat & fish	28.9
**2**	Human milk	7.4	Meat & fish	17.7	Cow’s milk	16.6
**3**	Meat & fish	7.2	Breads, pita, saj	6.1	Breads, pita, saj	7.0
**4**	Baby cereals	6.6	Dairy products	4.7	Dairy products	5.3
**5**	Rice & pasta	2.7	Rice & pasta	4.2	Vegetables (non-baby food)	3.9
**6**	Dairy products	2.6	Vegetables (non-baby food)	3.3	Butter & animal fats	3.7
**7**	Breads, pita, saj	2.4	Beans and legumes	3.1	Rice & pasta	3.6
**8**	Beans and legumes	2.2	Cow’s milk	2.1	Sweet bakery	3.4
**9**	Cow’s milk	2.1			Peanut butter, nuts, and seeds	3.1
**10**					Beans and legumes	3.0
**11**					Potatoes (non-baby food)	2.5
**12**					Potato chips	2.4
**Top contributors**:		**89.4**		**82.3**		**83.3**

### Calcium

In terms of calcium inadequacy relative to the EAR, the greatest prevalence was observed among toddlers (44.6%) and preschoolers (35.9%), compared to 8.8% among infants. I/YC formula was the number one contributor to calcium intakes among infants and toddlers, providing around 49.8% and 43.5% of calcium intake, respectively. While human milk was ranked as the second major contributor to calcium intake among infants (11.9%), this contribution decreased to 2.4% among toddlers. The contribution of cow’s milk and dairy products to calcium intakes gradually increased from 6.2% and 7.5% among infants to 41.4% and 22.1% among preschoolers, respectively. Other important contributors to calcium intakes included butter and animal fats (which contributed to 7.5–9.2% depending on the age group) and vegetables (contributing to around 3.1–3.7% among toddlers and preschoolers, respectively) (Table 5).

**Table 5:** Main food contributors and their percent contribution to calcium intake among infants and young children by age group in Lebanon, 2012

% exceeding AI/EAR	Age 6-11.9 m *n* = 148		Age 12-23.9 m *n* = 222		Age 24-47.9 m *n* = 393	
	91.2% > AI		55.4% > EAR		64.1% >EAR	
Rank	Food group	% contribution	Food group	% contribution	Food group	% contribution
**1**	Infant/young child (I/YC) formula	49.8	Infant/young child (I/YC) formula	43.5	Cow’s milk	41.4
**2**	Human milk	11.9	Dairy products	18.1	Dairy products	22.1
**3**	Baby cereals	7.8	Butter & animal fats	9.2	Butter & animal fats	8.3
**4**	Dairy products	7.5	Cow’s milk	6.9	Vegetables (non-baby food)	3.7
**5**	Butter & animal fats	7.5	Vegetables (non-baby food)	3.1	Sweet bakery	3.7
**6**	Cow’s milk	6.2	Human milk	2.4	Ice cream, frozen yogurt, puddings	3.0
**7**					Herbs & seasonings	2.6
**8**					Sweetened beverages	2.3
**Top contributors**:		**90.6**		**83.3**		**87.1**

### Vitamin D

**Fig. 1:**
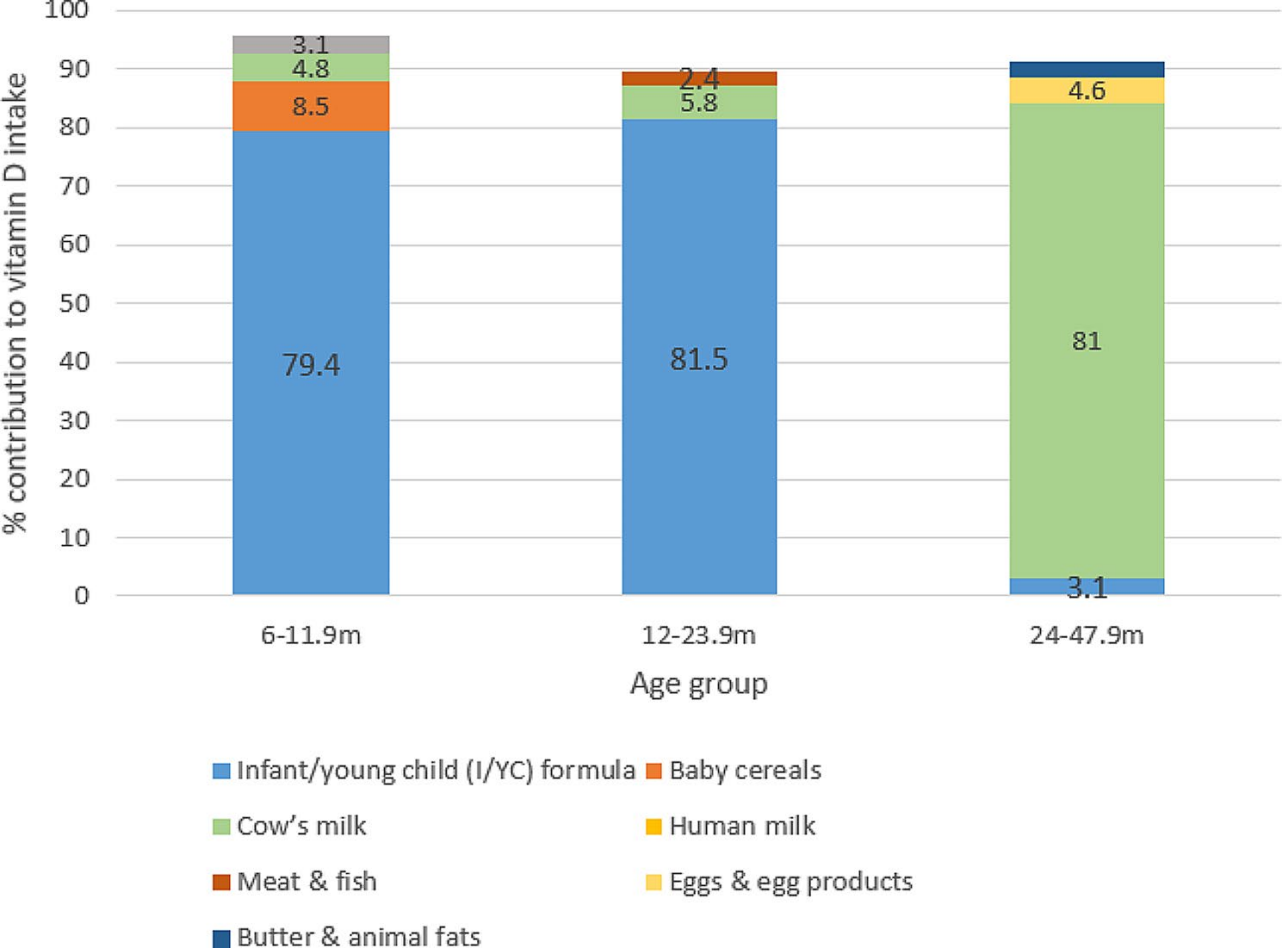
Main food contributors and their percent contribution to vitamin D intake among infants and young children by age group in Lebanon, 2012

It was estimated that 21.6% of infants achieved the AI for vitamin D, and this proportion decreased to 15.3% and 8.1% among toddlers and preschoolers, respectively (data not shown). I/YC formula and cow’s milk were the major contributors to vitamin D intakes across the three age groups. Baby cereals contributed to 8.5% of vitamin D intake among infants, meat and fish provided 2.4% of vitamin D intake among toddlers, and eggs and egg products provided 4.6% of the intake among preschoolers (Fig. 1).

### Vitamin A

Approximately 39% of infants had vitamin A intakes above the AI level, while 87.4% of toddlers and 64.9% of preschoolers exceeded the EAR. The top-ranking source of vitamin A among infants and toddlers was I/YC formula, providing 59.0% and 54.8%, respectively. This contribution decreased to 2.2% among preschoolers whose major contributor to vitamin A intake was cow’s milk (providing 25.2% of total vitamin A intake). The contribution of vegetables to vitamin A intake increased from 6.6% in infants to 11.5% in toddlers and 22.1% in preschoolers. Other important contributors to vitamin A intake among toddlers and preschoolers were animal products such as butter and animal fats, meat and fish, as well as dairy products. The contribution of breakfast cereals to vitamin A intakes was estimated to range between 2.3% and 4.2% among toddlers and preschoolers, respectively (Table 6).

**Table 6:** Main food contributors and their percent contribution to vitamin A intake among infants and young children by age group in Lebanon, 2012

% exceeding AI/EAR	Age 6-11.9 m *n* = 148		Age 12-23.9 m *n* = 222		Age 24-47.9 m *n* = 393	
	39.2% > AI		87.4% > EAR		64.9% > EAR	
Rank	Food group	% contribution	Food group	% contribution	Food group	% contribution
**1**	Infant/young child (I/YC) formula	59.0	Infant/young child (I/YC) formula	54.8	Cow’s milk	25.2
**2**	Human milk	23.3	Vegetables (non-baby food)	11.5	Vegetables (non-baby food)	22.1
**3**	Vegetables (non-baby food)	6.6	Meat & fish	7.5	Meat & fish	9.1
**4**			Butter & animal fats	5.0	Butter & animal fats	8.1
**5**			Dairy products	2.8	Eggs & egg products	4.8
**6**			Melon	2.5	Ice cream, frozen yogurt, puddings	4.6
**7**			Breakfast cereals	2.3	Dairy products	4.4
**8**					Breakfast cereals	4.2
**9**					Melon	3.7
**10**					Sweet bakery	3.6
**11**					Sweetened beverages	2.8
**12**					Infant/young child (I/YC) formula	2.2
**Top contributors** :		**88.9**		**88.6**		**94.7**

### Folate

More than 80% of infants exceeded the AI level for folate, while close to 80% and 66% of toddlers and preschoolers had intakes above the EAR levels, respectively. The top three ranking food sources of folate among infants and toddlers were I/YC formula, rice & pasta, and beans and legumes. In specific, the contribution to folate intake was of 49.7% and 36.6% from I/YC formula, 9.1% and 11.5% from rice & pasta, and 7.9% and 10.7% from beans and legumes among infants and toddlers, respectively. The contribution of beans and legumes to folate intake increased to 12% to become the top contributor to folate intake among preschoolers, followed by vegetables and rice and pasta which contributed to around 11% and 9% of folate intake in this age group, respectively. (Table 7).

**Table 7:** Main food contributors and their percent contribution to folate intake among infants and children by age group in Lebanon, 2012

% exceeding AI/EAR	Age 6-11.9 m *n* = 148		Age 12-23.9 m *n* = 222		Age 24-47.9 m *n* = 393	
	80.4% >AI		79.7% >EAR		66.2% >EAR	
Rank	Food group	% contribution	Food group	% contribution	Food group	% contribution
**1**	Infant/young child (I/YC) formula	49.7	Infant/young child (I/YC) formula	36.6	Beans and legumes	12.0
**2**	Rice & pasta	9.1	Rice & pasta	11.5	Vegetables (non-baby food)	11.4
**3**	Beans and legumes	7.9	Beans and legumes	10.7	Rice & pasta	9.4
**4**	Baby cereals	6.3	Vegetables (non-baby food)	8.0	Sweetened beverages	7.8
**5**	Human milk	5.7	Sweet bakery	5.4	Sweet bakery	6.8
**6**	Sweet bakery	4.1	Breakfast cereals	4.1	Breakfast cereals	6.4
**7**	Vegetables (non-baby food)	3.9	Sweetened beverages	2.7	Cow’s milk	6.1
**8**			Breads, pita, saj	2.6	100% juices	5.6
**9**					Meat & fish	4.8
**10**					Potatoes (non-baby food)	4.3
**11**					Breads, pita, saj	3.1
**12**					Crackers, rice cakes, kaak	2.9
**13**					Herbs & seasonings	2.6
**14**					Eggs & egg products	2.1
**Top contributors** :		**86.7**		**81.6**		**85.3**

## Discussion

The findings of this study provide a comprehensive picture of the major dietary sources of fiber and several micronutrients of concern among infants and young children in Lebanon and an insight into the changes in food consumption patterns with age.

In line with findings reported by other studies [26], fiber intakes were far below the recommendations in our study sample although beans and legumes, vegetables, and fruits were identified as the top sources of fiber for infants and young children aged 6-47.9 months. Given that the ranking of a food as a source of nutrient reflects not only the concentration of a nutrient in a food but also the frequency of consumption of the food, the low intake of fiber can be explained by the fact that these food groups rich in fiber were not being consumed in sufficient amounts. In fact, a previous study conducted by our group on the same population showed that adherence to fruits and vegetables recommendations was low in those aged 12-47.9 months [5]. The present study has also identified that the contribution of fruits to fiber intake decreased with older age groups. These findings are consistent with those reported by the FITS study in the United Arab Emirates [27] where fruits had a greater percent contribution to fiber intake among infants as compared to preschoolers. However, unlike findings reported from United Arab Emirates [27], our study findings indicate a low contribution of grains to fiber intakes in Lebanese infants and young children and this may at least partially explain the fact that a small percentage of toddlers and preschoolers met the fiber AI levels. It is important to note here that grains were consumed in their refined, low fiber forms, and only 54 children of the study participants (7.1%) had reported whole grain consumption (data not shown). These findings, therefore, highlight the importance of initiating complementary feeding practices that focus on promoting adequate fiber intake through the consumption fruits, vegetables, beans and legumes, as well as whole-grains. The adoption of a high fiber grain-based diet would be a practical and energy-neutral strategy for increasing fiber intakes as well as closing the gap between fiber intakes and fiber recommendations. This has been actually proposed by a group of nutrition researchers, educators, and communicators during the roundtable discussions “Filling America’s Fiber Gap: Probing Realistic Solutions” [28], given that the Dietary Guidelines for Americans 2015–2020 has recommended that at least half of the grains be consumed as whole grains [29].

This study has also observed that I/YC formula and cow’s milk constitute major food sources of vitamin D among children aged 6-47.9 months. However, the prevalence of vitamin D inadequacy is still high among these children. A previous study conducted on the same population has shown that only half of the children are adhering to the AHA/AAP Dietary Recommendations for milks and dairy [5], and hence more efforts ought to be made in promoting adequate milk/dairy intake in these age groups. In fact, several studies have showed that adequate intake of milk and dairy products is associated with improved bone health, reduction of the risk of developing type 1 diabetes, and long-term programming of the immune response pattern [30]. Among infants, cow’s milk was found to contribute substantially to zinc, calcium, and vitamin D intakes. Similar findings were reported by a study conducted among infants and children in Mexico [23]. This is of concern given that cow’s milk introduction before the age of 1 year can lead to cow’s milk allergy and iron-deficiency anemia [31]. It is, therefore, very important to encourage mothers to transition to iron-fortified formula rather than cow’s milk, if the optimal food in infancy, which is human breast milk, is not available.

Of concern also is the finding that sweetened beverages and sweet bakery were among the top food sources contributing to different nutrients intakes, including fiber, iron, zinc, calcium, vitamin A, and folate among the three age groups. Contribution of sweet bakery (which includes traditional sweets, cakes, pies, biscuits, cookies, bars, brownies, and muffins) to fiber, iron, and folate was even observed among infants, and this is aligned with similar observations from different countries, namely Philippines [32], Mexico [23], United States [33], China [34], and United Arab Emirates [27]. Early consumption of sweetened food and/or beverages and the high contribution of these foods to different nutrients at an older age (24-47.9 months) is another alarming finding in this study. For example, sweet bakery was the top contributor to iron intake and the fifth contributor to fiber, calcium, and folate intakes. As they grow, infants experience physiological shifts in nutrient and energy requirements that can no longer be supported by breast milk alone [32]. Therefore, promoting intakes of complementary foods that are nutrient-dense, i.e., relatively low in calories and high in vitamins and minerals, in lieu of sweet bakery and sweetened beverages may be one strategy to improve dietary intakes of nutrients and diet quality in this population and thus establish healthy eating habits from early childhood [35].

Our findings with regards to the high contribution of I/YC formula and baby cereals to iron, zinc, calcium, vitamin D, vitamin A (no significant contribution from baby cereals), as well as folate intakes among infants and toddlers align with what was found among children in the United Arab Emirates [27], South Africa [36], Mexico [23], and France [37]. The percent contribution of baby cereals to these nutrients was higher among infants than in toddlers and this can be attributed to the fact that baby cereals are the first foods to be introduced to infants in countries of the MENA region, including Lebanon [38]. Moreover, I/YC formula and baby cereals are fortified food sources, and this explains their important contribution to these nutrients’ intakes. Previous studies have shown that the consumption of fortified food products may be an effective strategy in improving nutrient intakes amongst children [39–42]. Bread is also another staple food source that is consumed in large quantities in the region and for which wheat flour fortification would be a simple, inexpensive and effective strategy for supplying vitamins and minerals to the diets of large segments of the region’s population [43]. However, all flour in Lebanon is still produced in industrial mills and none is fortified as Lebanon does not have mandatory or voluntary wheat flour fortification [43]. The difficulty in harmonizing standards and procedures for inspection, and coordinating the involvement of three government ministries; an inactive national coordination committee on wheat flour fortification; the high cost of the proposed measures; and the absence of testing for iron in wheat flour are all challenges for wheat flour fortification in Lebanon [43]. These challenges should be addressed to implement and formulate relevant legislations of fortification in Lebanon [43, 44], knowing that the Flour Fortification Initiative revealed that all Middle Eastern countries have invested in mandatory micronutrient fortification of wheat flour for locally manufactured and imported products with the exception of Lebanon [44].

Amongst toddlers and preschoolers, meat and fish ranked as a major contributor to iron intakes. This is an important finding, given that regular consumption of animal-based foods can prevent a decrease in hemoglobin in late infancy [45]. However, attention should be warranted for the inclusion of lean red meats as part of a healthy, varied diet during this critical phase of children’s development process, especially that earlier studies conducted in Lebanon have highlighted that, with the ongoing nutrition transition, the intakes of meat and poultry are following an increasing trend [46]. At the same time, this finding highlights concerns about the affordability of lean red meat, underscoring the need for targeted interventions to ensure that lean meat remain economically accessible to families facing financial constraints, particularly after the pressing economic, political, environmental, and health constraints that Lebanon has been witnessing [47]. In line with findings reported in the FITS study conducted in United Arab Emirates [27], herbs and seasonings have also contributed markedly to iron intakes among preschoolers. This is mainly contributed by dried thyme (za’atar) followed by dried spearmint, both of which are local herbs that are widely consumed in Lebanon in salads or as a spread on bread for dried thyme (za’atar) when mixed with olive oil [48]. It is worth noting that though dried thyme leaves are particularly rich in iron (117.2 mg/100 g of dry matter), a study has revealed that the bioavailability of iron in thyme is low [49].

### Strengths and limitations

This study is a nationally representative, cross-sectional study of infants and children in Lebanon. To our knowledge, this is the only analysis to date that specifically explores the top sources of key nutrients among 3 distinct age groups of infants and children, allowing us to better capture the changes in food consumption practices that occur in early life. However, the results of this study ought to be interpreted in light of the following limitations. This study was a cross-sectional in design, so it was not possible to assess the longitudinal changes in the food sources of key micronutrients with time among the same individuals. Dietary intake assessment relies on a single 24-HR, which may not reflect usual intakes. Therefore, further studies should include more than one 24-HR, at least for a subsample of the population. Despite the well-acknowledged limitations of the 24-HR approach, such as reliance on memory and possible day-to-day variation, the 24-HR may provide accurate estimates of energy intake at the population level, especially that dietary data was collected by the multiple pass approach in this study [50, 51]. Well-trained research nutritionists administered all recalls in order to attenuate interviewer errors. Another limitation is the use of the USDA database in nutrient intake estimation, given the lack of food composition databases that are specific to Lebanon or even the region. The USDA database may not correctly capture the nutrient composition of local food varieties in Lebanon and may thus represent a source of error in intake estimations [52]. In addition, the USDA database does not include many of the composite traditional dishes consumed in the country. In order to address this limitation and be able to assess nutrient intakes from mixed traditional dishes, we have added standardized recipes to the Nutritionist Pro software using single food items [52]. Moreover, dietary intakes of micronutrients considered for this study reflect intakes from diet only and dietary supplement intakes were not accounted for. Future research in Lebanon should assess supplement use and its impact on nutrient intake and adequacy among this population. Finally, given that the survey was conducted in 2012, its findings may underestimate the effects of the nutrition transition on dietary intakes in young children.

## Conclusions

In conclusion, this study showed that a substantial proportion of Lebanese infants and young children had inadequate intakes of key nutrients, including fiber, iron, zinc, calcium, vitamin D, vitamin A, and folate. It also identified the main food sources for these nutrients, by age group. The results showed that in addition to milk sources, vegetables, beans and legumes, breads, meats, and rice and pasta, sweet bakery and sweetened beverages have contributed to nutrients intakes from early ages. This calls for implementing initiatives and designing approaches to support nutrition education and improve nutrient intakes in infancy and early childhood, and hence improve the nutritional status of this population group.

## Data Availability

The datasets used and/or analyzed during the current study are available from the corresponding author on reasonable request.

## Abbreviations

FITS: Feeding Infants and Toddlers Study
ELNAHL: Early Life Nutrition and Health in Lebanon
USDA: United States Department of Agriculture
24-HR: Multiple Pass 24- Hour Dietary Recall
AUB: American University of Beirut
DRIs: Dietary Reference Intakes
EAR: Estimated Average Requirement
AI: Adequate Intake
SSBs: Sugar-sweetened beverages
I/YC Formula: Infant/young child formula
